# Supplementary material for: Q-Herilearn: Assessing heritage learning in digital environments. A mixed approach with factor and IRT models
Source: PLoS One. 2024 Mar 29;19(3):e0299733. doi: 10.1371/journal.pone.0299733 (PMC10980239; doi:10.1371/journal.pone.0299733)
Supplement: S1 Table — (DOCX) [file pone.0299733.s001.docx]

#### The content of the final items (formulated in both English and Spanish) is shown in Tables 1 to 7 (in italics, final items).

| **S1 Table. Knowing dimension.** | | |
| --- | --- | --- |
| **Item** | **Contents** |  |
| *Kno001* | *I learn new things about heritage in digital environments* | *Aprendo cosas nuevas sobre el patrimonio en entornos digitales.* |
| Kno002 | I make use of digital geolocation tools to identify places of heritage interest | Hago uso de herramientas digitales de geolocalización para identificar lugares de interés patrimonial. |
| Kno003 | I am interested in the applications aimed at the knowledge of cultural heritage. | Me intereso por las aplicaciones dirigidas al conocimiento del patrimonio cultural. |
| *Kno004* | *The digital environment allows to know about heritage through images.* | *El entorno digital permite conocer patrimonios a través de imágenes.* |
| Kno005 | In digital environments I contrast my own experience with the experience of other users in relation to heritage. | En los entornos digitales contrasto mi propia experiencia con la de otros usuarios en relación con el patrimonio. |
| *Kno006* | *What I see in a digital environment encourages me to keep looking for other heritages.* | *Lo que veo en un entorno digital me anima a seguir buscando otros patrimonios.* |
| Kno007 | To obtain information about a heritage property I use digital environments. | Para obtener información sobre un bien patrimonial utilizo entornos digitales. |
| Kno008 | Browsing a museum's website helps me to temporarily locate the most important pieces of the museum's collection. | Navegar por la web de un museo ayuda a ubicar temporalmente sus piezas más destacadas. |
| *Kno009* | *I look for social networks that help me to learn more about heritage.* | *Busco redes sociales que me ayuden a conocer más sobre patrimonio.* |
| *Kno010* | *I read online news about heritage.* | *Leo noticias en red sobre patrimonio.* |
| *Kno011* | *I look for information when a post about heritage arouses my curiosity for further information.* | *Busco información cuando un post sobre patrimonio me despierta curiosidad de ampliación.* |
| Kno012 | Reading the opinions and comments of other users allows me to increase the information I have about heritage sites. | Leer las opiniones y comentarios de otros usuarios me permite aumentar la información que tengo sobre los patrimonios. |
| *Kno013* | *I access digital environments to learn about new heritages.* | *Accedo a entornos digitales para conocer nuevos patrimonios.* |
| Kno014 | Viewing the publications of other users allows me to expand my knowledge about heritage. | Visualizar las publicaciones de otros usuarios me permite ampliar mis conocimientos sobre patrimonio. |
