# Supplementary material for: Q-Herilearn: Assessing heritage learning in digital environments. A mixed approach with factor and IRT models
Source: PLoS One. 2024 Mar 29;19(3):e0299733. doi: 10.1371/journal.pone.0299733 (PMC10980239; doi:10.1371/journal.pone.0299733)
Supplement: S2 Table — (DOCX) [file pone.0299733.s002.docx]

#### The content of the final items (formulated in both English and Spanish) is shown in Tables 1 to 7 (in italics, final items).

| **S2 Table. Understanding dimension.** | | |
| --- | --- | --- |
| **Item** | **Contents** |  |
| *Und015* | *The digital environment allows me to understand the heritage using maps.* | *El entorno digital permite comprender el patrimonio utilizando mapas.* |
| Und016 | The digital environment makes me understand the relationship between heritage and history. | El entorno digital hace que comprenda la relación entre patrimonio e historia. |
| *Und017* | *The images of the digital environment help me understand the heritage.* | *Las imágenes del entorno digital ayudan a entender el patrimonio.* |
| Und018 | I understand heritage through testimonies in digital environments. | Comprendo el patrimonio a través de testimonios en entornos digitales. |
| Und019 | The images of the digital environment show me details to better understand each heritage treated. | Las imágenes del entorno digital me muestran detalles para entender mejor cada patrimonio tratado. |
| *Und020* | *The audios in the digital environment help me to understand the heritage they deal with.* | *Los audios del entorno digital me ayudan a entender el patrimonio del que tratan.* |
| *Und021* | *When I need clarifications about heritage assets I resort to the use of digital environments.* | *Cuando necesito aclaraciones sobre bienes patrimoniales recurro al uso de entornos digitales.* |
| *Und022* | *The review of experiences published in the heritage social networks helps me to understand the heritage.* | *La revisión de experiencias publicadas en las RRSS patrimoniales favorecen la comprensión del patrimonio.* |
| *Und023* | *Virtual reality and augmented reality are means that help me better understand cultural heritage.* | *La realidad virtual y la realidad aumentada son medios que me ayudan a comprender mejor el patrimonio cultural.* |
| *Und024* | *3D recreation allows understanding the dimensions of an ancient settlement/village and what its streets and buildings were like.* | *La recreación 3D permite comprender las dimensiones de un antiguo poblado/villa y cómo eran sus calles y edificios.* |
|  |  |  |
