# Supplementary material for: Q-Herilearn: Assessing heritage learning in digital environments. A mixed approach with factor and IRT models
Source: PLoS One. 2024 Mar 29;19(3):e0299733. doi: 10.1371/journal.pone.0299733 (PMC10980239; doi:10.1371/journal.pone.0299733)
Supplement: S3 Table — (DOCX) [file pone.0299733.s003.docx]

#### The content of the final items (formulated in both English and Spanish) is shown in Tables 1 to 7 (in italics, final items).

| **S3 Table. Respecting dimension.** | | |
| --- | --- | --- |
| **Item** | **Contents** |  |
| Res025 | The digital environment fosters respect for different heritages. | El entorno digital fomenta el respeto por los diferentes patrimonios. |
| *Res026* | *The digital environment raises awareness of the risks that can threaten heritage assets.* | *El entorno digital permite concienciar sobre los riesgos que pueden amenazar a los bienes patrimoniales.* |
| Res027 | The digital environment presents an inclusive vision with attention to minorities. | El entorno digital presenta una visión integradora con atención a las minorías. |
| Res028 | The digital environment gathers user initiatives for heritage protection. | El entorno digital recoge iniciativas del usuario para la protección del patrimonio. |
| *Res029* | *I urge others to be respectful with any typology of cultural heritage.* | *Insto al resto a ser respetuosos con cualquier tipología de patrimonio cultural.* |
| *Res030* | *I have an attitude of respect towards the diversity of personal heritages.* | *Tengo una actitud de respeto hacia la diversidad de patrimonios personales.* |
| Res031 | Digital environments contribute to perpetuate respectful attitudes about heritage. | Los entornos digitales contribuyen a perpetuar actitudes de respeto en torno al patrimonio. |
| *Res032* | *Digital environments specific to heritage contribute to perpetuate respectful attitudes about heritage.* | *Los entornos digitales específicos sobre patrimonio contribuyen a perpetuar actitudes de respeto en torno al mismo.* |
| *Res033* | *People can express different opinions about heritage in digital environments.* | *Las personas podemos expresar opiniones diferentes sobre el patrimonio en los entornos digitales.* |
| *Res034* | *Digital environments foster caring attitudes towards heritage.* | *En los entornos digitales se fomentan actitudes de cuidado hacia el patrimonio.* |
| Res035 | In digital environments the need for heritage care is conveyed. | En los entornos digitales se transmite la necesidad del cuidado del patrimonio. |
| *Res036* | *I respect all heritage assets even if their origin and meaning does not agree with my ideology.* | *Respeto todos los bienes patrimoniales aunque su origen y significado no concuerde con mi ideología.* |
