# Supplementary material for: Q-Herilearn: Assessing heritage learning in digital environments. A mixed approach with factor and IRT models
Source: PLoS One. 2024 Mar 29;19(3):e0299733. doi: 10.1371/journal.pone.0299733 (PMC10980239; doi:10.1371/journal.pone.0299733)
Supplement: S4 Table — (DOCX) [file pone.0299733.s004.docx]

#### The content of the final items (formulated in both English and Spanish) is shown in Tables 1 to 7 (in italics, final items).

| **S4 Table. Valuing dimension.** | | |
| --- | --- | --- |
| **Item** | **Contents** |  |
| Val037 | I change my assessment of a heritage work after consulting a heritage-centered application. | Cambio de valoración sobre una obra patrimonial después de consultar una aplicación centrada en el patrimonio. |
| Val038 | The digital environment enhances the value of heritage with gender perspective. | El entorno digital pone en valor el patrimonio con perspectiva de genero. |
| Val039 | I value the heritage element as part of my history. | Valoro el elemento patrimonial como parte de mi historia. |
| Val040 | I value the heritage element as a part of my culture. | Valoro el elemento patrimonial como parte de mi cultura. |
| Val041 | After using a digital environment I feel that the heritage it deals with is important. | Tras usar un entorno digital siento que el patrimonio del que trata es importante. |
| Val042 | I recognize the value that heritage has for my community. | Reconozco el valor que tiene el patrimonio para mi comunidad. |
| *Val043* | *I generate links with the cultural heritage worked on in the digital environment.* | *Genero vínculos con el patrimonio cultural trabajado en el entorno digital.* |
| Val044 | Use the option "like" in RRSS when show content related to the heritage. | Utilizo la opción “like” en RRSS cuando muestran contenido relacionado con el patrimonio. |
| *Val045* | *The use of digital environments helps me to better assess the cultural heritage I visit.* | *El uso de entornos digitales me ayuda a valorar mejor el patrimonio cultural que visito.* |
| *Val046* | *The presence of heritage-related content in the main RRSS helps the heritage is valued by the community.* | *La presencia de contenidos patrimoniales en las principales RRSS ayuda a que el patrimonio sea valorado por la comunidad.* |
| Val047 | I mention the importance of heritage in forums or social networks. | Menciono la importancia del patrimonio en foros o redes sociales. |
| *Val048* | *The digital environment allows me to compare my patrimonial experiences with others.* | *El entorno digital me permite comparar mis experiencias patrimoniales con las de los demás.* |
| *Val049* | *The digital environment allows me to value heritage experiences of others.* | *El entorno digital me permite valorar experiencias patrimoniales de otros.* |
| *Val050* | *You can discover the value of the heritage participating in APPs collaborative on cultural assets.* | *Se puede descubrir el valor del patrimonio participando en APPs colaborativas sobre bienes culturales.* |
| *Val051* | *Sharing heritage on the network increases my perception of its value.* | *Compartir patrimonios en red aumenta mi percepción sobre su valor.* |
