# Supplementary material for: Q-Herilearn: Assessing heritage learning in digital environments. A mixed approach with factor and IRT models
Source: PLoS One. 2024 Mar 29;19(3):e0299733. doi: 10.1371/journal.pone.0299733 (PMC10980239; doi:10.1371/journal.pone.0299733)
Supplement: S5 Table — (DOCX) [file pone.0299733.s005.docx]

#### The content of the final items (formulated in both English and Spanish) is shown in Tables 1 to 7 (in italics, final items).

| **S5 Table. Caring dimension.** | | |
| --- | --- | --- |
| **Item** | **Contents** |  |
| Car052 | I participate in civil society initiatives on patrimonial maintenance and conservation. | Participo en iniciativas de la sociedad civil sobre el mantenimiento y conservación patrimonial. |
| Car053 | I participate in digital initiatives for the safeguarding of heritage assets. | Participo en iniciativas digitales para la salvaguarda de bienes patrimoniales. |
| Car054 | I am concerned about the destruction of heritage during armed conflicts. | Me preocupa la destrucción del patrimonio durante conflictos armados. |
| Car055 | I participate in heritage safeguarding campaigns in digital environments. | Participio en campañas de salvaguarda del patrimonio en entornos digitales. |
| *Car056* | *I denounce in RRSS actions that aim to destroy heritage elements.* | *Denuncio en RRSS acciones que pretenden acabar con elementos patrimoniales.* |
| *Car057* | *I make visible in social networks places that are deteriorated in order to raise awareness about the care of the assets.* | *Visibilizo en las RRSS lugares que están deteriorados para concienciar sobre el cuidado de los bienes.* |
| *Car058* | *I participate in forums for the safeguarding of heritage to fight for the care of assets.* | *Participo en foros de salvaguarda del patrimonio para luchar por el cuidado de los bienes.* |
| *Car059* | *I share news on the network about heritage conservation that can help other people to learn about ways to care for them.* | *Comparto noticias en la red sobre la conservación del patrimonio que pueda ayudar a otras personas a conocer las formas de cuidado.* |
| *Car060* | *I collaborate in action networks for the protection of heritage and the dangers of not taking care of it.* | *Colaboro en redes de acción por la protección del patrimonio y los peligros de no cuidarlo.* |
| Car061 | Knowing other users' heritages increases my interest in their conservation. | Conocer los patrimonios de otros usuarios aumenta mi interés en su conservación. |
| Car062 | The contact with the heritage in digital environments incites me to take care of it. | El contacto con el patrimonio en entornos digitales me incita a cuidarlo. |
| *Car063* | *I use digital environments so that the heritages of my surroundings are not lost / forgotten.* | *Utilizo entornos digitales para que los patrimonios de mi entorno no se pierdan / olviden.* |
| *Car064* | *I feel the need to protect the heritages of my environment through digital environments.* | *Siento la necesidad de proteger los patrimonios de mi entorno a través de entornos digitales.* |
