# Supplementary material for: Q-Herilearn: Assessing heritage learning in digital environments. A mixed approach with factor and IRT models
Source: PLoS One. 2024 Mar 29;19(3):e0299733. doi: 10.1371/journal.pone.0299733 (PMC10980239; doi:10.1371/journal.pone.0299733)
Supplement: S6 Table — (DOCX) [file pone.0299733.s006.docx]

#### The content of the final items (formulated in both English and Spanish) is shown in Tables 1 to 7 (in italics, final items).

| **S6 Table. Enjoying dimension.** | | |
| --- | --- | --- |
| **Item** | **Contents** |  |
| Enj065 | Sharing my vision of a heritage asset and receiving feedback increases my satisfaction. | Compartir mi visión sobre un bien patrimonial y recibir feedback aumenta mi satisfacción. |
| Enj066 | I enjoy when I read curiosities about cultural heritage. | Disfruto cuando leo curiosidades sobre el patrimonio cultural. |
| *Enj067* | *I am excited to discover heritage content on the Internet, social networks or app.* | *Me hace ilusión descubrir contenidos sobre patrimonio en Internet, redes sociales o app.* |
| Enj068 | I feel good when I discover in digital media artistic and heritage works that I didn't know about. | Me siento bien cuando descubro en medios digitales obras artísticas y patrimoniales que no conocía. |
| Enj069 | I like to publish photos of heritage elements in digital environments. | Me gusta publicar fotos de elementos patrimoniales en entornos digitales. |
| Enj070 | I enjoy when I integrate myself in virtual heritage communities. | Disfruto cuando me integro en comunidades patrimoniales virtuales. |
| *Enj071* | *The videos of the digital environment make me enjoy the heritage.* | *Los videos del entorno digital hacen que disfrute del patrimonio.* |
| Enj072 | I enjoy learning about other people's heritage. | Me gusta conocer el patrimonio de otras personas. |
| Enj073 | I enjoy watching informative heritage videos in RRSS or APPs (Educaplay-memes). | Disfruto viendo vídeos informativos de patrimonio en RRSS o APPs (Educaplay-memes). |
| *Enj074* | *I like to visualize recreations of heritage elements.* | *Me gusta visualizar recreaciones de elementos patrimoniales.* |
| Enj075 | I like to take photographs of the heritage environment. | Me gusta fotografiar el entorno patrimonial. |
| *Enj076* | *In my spare time, I follow specific heritage accounts.* | *En mis ratos de ocio, sigo cuentas específicas de patrimonio.* |
| *Enj077* | *I really like to imagine other times and other places thanks to digital and audiovisual content related to heritage.* | *Me gusta mucho imaginar otras épocas y otros lugares gracias a los contenidos digitales y audiovisuales vinculados al patrimonio.* |
| Enj078 | I enjoy when I read heritage life stories in APPs or RRSS. | Disfruto cuando leo historias de vida patrimoniales en APPs o RRSS. |
| Enj079 | I enjoy following museum pages on my social network of reference. | Disfruto siguiendo páginas de museos en mi red social de referencia. |
| *Enj080* | *I am excited to see heritage from my region shared by other people.* | *Me emociona ver patrimonio de mi región compartido por otras personas.* |
| *Enj081* | *Knowing other visions about a heritage helps me to enjoy it deeply.* | *Conocer otras visiones sobre un patrimonio me ayuda a disfrutarlo en profundidad.* |
