# Supplementary material for: Q-Herilearn: Assessing heritage learning in digital environments. A mixed approach with factor and IRT models
Source: PLoS One. 2024 Mar 29;19(3):e0299733. doi: 10.1371/journal.pone.0299733 (PMC10980239; doi:10.1371/journal.pone.0299733)
Supplement: S7 Table — (DOCX) [file pone.0299733.s007.docx]

#### The content of the final items (formulated in both English and Spanish) is shown in Tables 1 to 7 (in italics, final items).

| **S7 Table. Transmitting dimension.** | | |
| --- | --- | --- |
| **Item** | **Contents** |  |
| Tra082 | Digital environments allow for immediate dissemination of possible attacks on heritage sites. | Los entornos digitales permiten una inmediata difusión de posibles atentados sobre el patrimonio. |
| Tra083 | I share photos of heritage sites to make them known when I go sightseeing. | Comparto fotos de lugares patrimoniales para darlos a conocer cuando hago turismo. |
| *Tra084* | *I favor the transmission of heritage when I share an element that has a special value for me.* | *Favorezco la transmisión del patrimonio cuando comparto un elemento que tiene un valor especial para mi.* |
| Tra085 | I share heritage in social networks through proposals from school or university education. | Comparto patrimonios en RRSS a través de propuestas desde la educación escolar o universitaria. |
| *Tra086* | *I communicate my interest in the heritage sites of my city in social networks.* | *Comunico mi interés por los lugares patrimoniales de mi ciudad en RRSS.* |
| *Tra087* | *I share photographs of my personal heritage in social networks.* | *Comparto fotografías de mi patrimonio personal en redes sociales.* |
| Tra088 | In my digital family environment, the dissemination of cultural, heritage and artistic elements is encouraged. | En mi entorno familiar digital, se fomenta la difusión de elementos culturales, patrimoniales y artísticos. |
| *Tra089* | *I encourage others to participate in heritage experiences that I find appealing.* | *Animo a otros a que participen en experiencias patrimoniales que me resultan atractivas.* |
| *Tra090* | *I recommend pages or applications related to heritage to my contacts.* | *Recomiendo a mis contactos páginas o aplicaciones vinculadas al patrimonio.* |
| Tra091 | Digital environments allow me to share the links that people establish with cultural assets. | Los entornos digitales me permiten compartir los vínculos que las personas establecen con los bienes culturales. |
| Tra092 | Giving my opinion about a heritage asset in a digital environment seems to me a good way to contribute to its transmission. | Dar mi opinión sobre un bien patrimonial en un entorno digital me parece un buen modo de contribuir a su transmisión. |
| Tra093 | Sharing images of a heritage property in a digital environment contributes to its survival. | Compartir imágenes de un bien patrimonial en un entorno digital contribuye a su pervivencia. |
| Tra094 | I belong to groups for the dissemination of artistic and heritage contents in social networks. | Pertenezco a grupos de difusión de contenidos artísticos y patrimoniales en redes sociales. |
| Tra095 | I share with acquaintances what I have learned through apps, RRSS or digital environments related to heritage. | Comento a conocidos lo aprendido a través de apps, RRSS o entornos digitales relacionados con el patrimonio. |
| *Tra096* | *I take photos with the intention of uploading them to apps, RRSS or digital environments related to heritage.* | *Saco fotos con la intención de subirlas a apps, RRSS o entornos digitales relacionados con el patrimonio.* |
| *Tra097* | *I participate in a social network or heritage-specific app by discussing or sharing content.* | *Participo en alguna red social o app específica de patrimonio debatiendo o compartiendo contenidos.* |
