# Supplementary material for: Q-Herilearn: Assessing heritage learning in digital environments. A mixed approach with factor and IRT models
Source: PLoS One. 2024 Mar 29;19(3):e0299733. doi: 10.1371/journal.pone.0299733 (PMC10980239; doi:10.1371/journal.pone.0299733)
Supplement: S8 Table — (DOCX) [file pone.0299733.s008.docx]

|  | | | | | | | | | | | | | | |
| --- | --- | --- | --- | --- | --- | --- | --- | --- | --- | --- | --- | --- | --- | --- |
| **S8 Table. Polychoric correlations.** | | | | | | | | | | | | | | |
|  | kno1 | kno4 | kno6 | kno9 | kno10 | kno11 | kno13 | und15 | und17 | und20 | und21 | und22 | und23 | und24 |
| kno1 | 1.000 |  |  |  |  |  |  |  |  |  |  |  |  |  |
| kno4 | .582 | 1.000 |  |  |  |  |  |  |  |  |  |  |  |  |
| kno6 | .603 | .698 | 1.000 |  |  |  |  |  |  |  |  |  |  |  |
| kno9 | .521 | .466 | .637 | 1.000 |  |  |  |  |  |  |  |  |  |  |
| kno10 | .598 | .420 | .554 | .597 | 1.000 |  |  |  |  |  |  |  |  |  |
| kno11 | .576 | .500 | .621 | .525 | .625 | 1.000 |  |  |  |  |  |  |  |  |
| kno13 | .614 | .576 | .710 | .658 | .656 | .645 | 1.000 |  |  |  |  |  |  |  |
| und15 | .413 | .554 | .561 | .381 | .411 | .484 | .523 | 1.000 |  |  |  |  |  |  |
| und17 | .521 | .590 | .590 | .424 | .484 | .538 | .572 | .576 | 1.000 |  |  |  |  |  |
| und20 | .423 | .428 | .495 | .454 | .426 | .455 | .503 | .454 | .675 | 1.000 |  |  |  |  |
| und21 | .433 | .508 | .482 | .314 | .310 | .455 | .446 | .451 | .533 | .424 | 1.000 |  |  |  |
| und22 | .439 | .468 | .536 | .473 | .417 | .502 | .520 | .498 | .595 | .571 | .544 | 1.000 |  |  |
| und23 | .321 | .416 | .424 | .346 | .344 | .355 | .408 | .399 | .501 | .481 | .415 | .507 | 1.000 |  |
| und24 | .338 | .461 | .435 | .200 | .248 | .371 | .365 | .431 | .533 | .412 | .443 | .480 | .645 | 1.000 |
| res26 | .447 | .425 | .417 | .313 | .398 | .411 | .390 | .376 | .505 | .435 | .399 | .496 | .404 | .426 |
| res29 | .299 | .334 | .339 | .242 | .317 | .370 | .283 | .311 | .387 | .295 | .332 | .378 | .313 | .362 |
| res30 | .303 | .379 | .354 | .115 | .258 | .384 | .229 | .308 | .390 | .229 | .424 | .359 | .274 | .395 |
| res32 | .373 | .380 | .403 | .259 | .285 | .386 | .340 | .283 | .493 | .394 | .386 | .459 | .365 | .417 |
| res33 | .195 | .329 | .236 | .064 | .135 | .279 | .190 | .236 | .328 | .214 | .356 | .313 | .247 | .332 |
| res34 | .306 | .324 | .302 | .254 | .235 | .324 | .272 | .241 | .396 | .331 | .307 | .379 | .305 | .350 |
| res36 | .217 | .270 | .262 | .079 | .162 | .298 | .183 | .208 | .318 | .199 | .333 | .237 | .229 | .324 |
| val43 | .407 | .373 | .459 | .389 | .403 | .438 | .420 | .377 | .485 | .423 | .305 | .455 | .365 | .372 |
| val45 | .401 | .443 | .457 | .319 | .377 | .433 | .386 | .385 | .550 | .437 | .460 | .478 | .430 | .466 |
| val46 | .343 | .382 | .371 | .306 | .315 | .399 | .317 | .312 | .449 | .367 | .439 | .428 | .370 | .408 |
| val48 | .353 | .391 | .447 | .364 | .297 | .411 | .409 | .394 | .419 | .392 | .379 | .460 | .381 | .407 |
| val49 | .361 | .399 | .442 | .365 | .317 | .423 | .406 | .345 | .452 | .411 | .377 | .507 | .349 | .423 |
| val50 | .304 | .362 | .373 | .278 | .261 | .330 | .330 | .298 | .412 | .397 | .303 | .452 | .422 | .410 |
| val51 | .345 | .394 | .438 | .371 | .311 | .382 | .402 | .308 | .472 | .424 | .363 | .471 | .436 | .399 |
| car56 | .264 | .190 | .286 | .358 | .315 | .314 | .320 | .233 | .256 | .305 | .153 | .305 | .265 | .220 |
| car57 | .222 | .216 | .305 | .365 | .327 | .277 | .364 | .212 | .279 | .322 | .124 | .316 | .314 | .230 |
| car58 | .215 | .118 | .261 | .369 | .350 | .242 | .354 | .183 | .209 | .282 | .062 | .221 | .214 | .072 |
| car59 | .302 | .238 | .332 | .416 | .434 | .357 | .394 | .246 | .303 | .377 | .168 | .334 | .317 | .220 |
| car60 | .235 | .163 | .316 | .393 | .369 | .301 | .416 | .223 | .217 | .337 | .099 | .289 | .259 | .138 |
| car63 | .370 | .345 | .431 | .440 | .439 | .382 | .475 | .339 | .347 | .393 | .269 | .380 | .353 | .273 |
| car64 | .391 | .362 | .431 | .482 | .430 | .384 | .474 | .318 | .418 | .426 | .260 | .384 | .345 | .292 |
| enj67 | .500 | .474 | .568 | .490 | .485 | .526 | .554 | .424 | .507 | .431 | .394 | .476 | .357 | .372 |
| enj71 | .438 | .437 | .506 | .415 | .443 | .484 | .465 | .406 | .504 | .468 | .398 | .465 | .411 | .406 |
| enj74 | .403 | .415 | .504 | .416 | .445 | .485 | .494 | .398 | .521 | .490 | .381 | .454 | .424 | .466 |
| enj76 | .484 | .353 | .507 | .599 | .574 | .476 | .551 | .339 | .437 | .402 | .253 | .384 | .279 | .236 |
| enj77 | .419 | .367 | .465 | .418 | .420 | .453 | .474 | .320 | .450 | .372 | .359 | .383 | .320 | .369 |
| enj80 | .386 | .394 | .483 | .399 | .437 | .462 | .462 | .321 | .441 | .357 | .373 | .432 | .321 | .371 |
| enj81 | .451 | .429 | .504 | .423 | .449 | .507 | .481 | .349 | .481 | .443 | .379 | .485 | .381 | .410 |
| tra84 | .343 | .394 | .440 | .410 | .347 | .401 | .414 | .374 | .395 | .338 | .355 | .406 | .389 | .337 |
| tra86 | .255 | .318 | .367 | .428 | .275 | .277 | .365 | .269 | .321 | .311 | .244 | .363 | .318 | .227 |
| tra87 | .222 | .270 | .305 | .352 | .208 | .242 | .324 | .236 | .283 | .266 | .242 | .353 | .303 | .225 |
| tra89 | .351 | .347 | .422 | .441 | .395 | .384 | .442 | .316 | .361 | .353 | .284 | .344 | .303 | .261 |
| tra90 | .372 | .299 | .411 | .509 | .444 | .368 | .476 | .279 | .351 | .379 | .189 | .379 | .321 | .220 |
| tra96 | .205 | .310 | .326 | .353 | .216 | .292 | .320 | .265 | .307 | .263 | .235 | .356 | .317 | .221 |
| tra97 | .258 | .200 | .288 | .395 | .331 | .236 | .368 | .183 | .280 | .300 | .091 | .267 | .271 | .096 |

| **S8 Table. Polychoric correlations.** (Cont.) | | | | | | | |  |  |  |  |  |  |  |  |
| --- | --- | --- | --- | --- | --- | --- | --- | --- | --- | --- | --- | --- | --- | --- | --- |
|  | res26 | res29 | res30 | res32 | res33 | res34 | res36 | | val43 | val45 | val46 | val48 | val49 | val50 | val51 |
| res26 | 1.000 |  |  |  |  |  |  | |  |  |  |  |  |  |  |
| res29 | .378 | 1.000 |  |  |  |  |  | |  |  |  |  |  |  |  |
| res30 | .418 | .633 | 1.000 |  |  |  |  | |  |  |  |  |  |  |  |
| res32 | .486 | .509 | .642 | 1.000 |  |  |  | |  |  |  |  |  |  |  |
| res33 | .409 | .339 | .569 | .494 | 1.000 |  |  | |  |  |  |  |  |  |  |
| res34 | .483 | .383 | .451 | .601 | .518 | 1.000 |  | |  |  |  |  |  |  |  |
| res36 | .306 | .459 | .688 | .461 | .509 | .379 | 1.000 | |  |  |  |  |  |  |  |
| val43 | .396 | .320 | .348 | .437 | .224 | .387 | .248 | | 1.000 |  |  |  |  |  |  |
| val45 | .446 | .385 | .491 | .524 | .409 | .424 | .397 | | .562 | 1.000 |  |  |  |  |  |
| val46 | .437 | .373 | .523 | .554 | .419 | .482 | .424 | | .465 | .631 | 1.000 |  |  |  |  |
| val48 | .386 | .278 | .330 | .433 | .339 | .358 | .276 | | .469 | .518 | .469 | 1.000 |  |  |  |
| val49 | .455 | .335 | .376 | .456 | .368 | .399 | .332 | | .460 | .538 | .545 | .738 | 1.000 |  |  |
| val50 | .384 | .306 | .354 | .459 | .321 | .406 | .271 | | .439 | .469 | .491 | .493 | .549 | 1.000 |  |
| val51 | .446 | .276 | .353 | .426 | .305 | .432 | .285 | | .470 | .513 | .520 | .542 | .606 | .580 | 1.000 |
| car56 | .284 | .271 | .072 | .198 | .048 | .173 | .015 | | .393 | .242 | .231 | .335 | .288 | .278 | .328 |
| car57 | .261 | .192 | .043 | .171 | .008 | .215 | -.064 | | .396 | .244 | .251 | .351 | .322 | .308 | .346 |
| car58 | .118 | .045 | -.135 | .086 | -.129 | .097 | -.187 | | .381 | .133 | .136 | .299 | .199 | .221 | .251 |
| car59 | .287 | .198 | .038 | .171 | .037 | .150 | -.042 | | .398 | .248 | .223 | .362 | .298 | .273 | .358 |
| car60 | .200 | .145 | -.041 | .140 | -.066 | .131 | -.110 | | .416 | .196 | .147 | .311 | .251 | .237 | .313 |
| car63 | .319 | .289 | .197 | .311 | .128 | .285 | .075 | | .474 | .364 | .315 | .424 | .387 | .359 | .422 |
| car64 | .376 | .290 | .225 | .340 | .137 | .330 | .141 | | .502 | .409 | .362 | .435 | .408 | .379 | .472 |
| enj67 | .438 | .362 | .412 | .427 | .261 | .368 | .291 | | .440 | .496 | .462 | .410 | .443 | .408 | .509 |
| enj71 | .414 | .357 | .385 | .429 | .261 | .371 | .309 | | .479 | .521 | .491 | .454 | .494 | .444 | .524 |
| enj74 | .414 | .382 | .368 | .450 | .214 | .369 | .299 | | .501 | .503 | .481 | .422 | .466 | .412 | .496 |
| enj76 | .311 | .223 | .119 | .270 | .036 | .216 | .048 | | .451 | .340 | .317 | .357 | .356 | .334 | .404 |
| enj77 | .307 | .359 | .302 | .412 | .222 | .310 | .236 | | .403 | .402 | .383 | .405 | .446 | .339 | .415 |
| enj80 | .398 | .380 | .415 | .434 | .314 | .365 | .326 | | .417 | .454 | .435 | .450 | .466 | .383 | .454 |
| enj81 | .449 | .415 | .451 | .466 | .315 | .403 | .350 | | .473 | .541 | .520 | .464 | .535 | .463 | .531 |
| tra84 | .346 | .359 | .351 | .346 | .214 | .310 | .273 | | .448 | .415 | .388 | .453 | .422 | .357 | .492 |
| tra86 | .252 | .253 | .185 | .276 | .147 | .240 | .097 | | .415 | .315 | .302 | .389 | .351 | .337 | .459 |
| tra87 | .206 | .190 | .131 | .209 | .145 | .185 | .072 | | .323 | .292 | .249 | .378 | .335 | .318 | .359 |
| tra89 | .277 | .275 | .207 | .300 | .145 | .248 | .123 | | .423 | .317 | .316 | .411 | .369 | .341 | .389 |
| tra90 | .294 | .199 | .068 | .237 | -.008 | .215 | .016 | | .432 | .280 | .241 | .354 | .329 | .319 | .366 |
| tra96 | .220 | .228 | .209 | .235 | .181 | .265 | .165 | | .353 | .322 | .298 | .359 | .351 | .326 | .408 |
| tra97 | .198 | .121 | -.047 | .140 | -.007 | .171 | -.114 | | .366 | .165 | .174 | .311 | .286 | .248 | .343 |

| **S8 Table. Polychoric correlations.** (Cont.) | | | | | | | | | | | | | | |
| --- | --- | --- | --- | --- | --- | --- | --- | --- | --- | --- | --- | --- | --- | --- |
|  | car56 | car57 | car58 | car59 | car60 | car63 | car64 | enj67 | enj71 | enj74 | enj76 | enj77 | enj80 | enj81 |
| car56 | 1.000 |  |  |  |  |  |  |  |  |  |  |  |  |  |
| car57 | .694 | 1.000 |  |  |  |  |  |  |  |  |  |  |  |  |
| car58 | .609 | .696 | 1.000 |  |  |  |  |  |  |  |  |  |  |  |
| car59 | .663 | .708 | .697 | 1.000 |  |  |  |  |  |  |  |  |  |  |
| car60 | .638 | .678 | .793 | .765 | 1.000 |  |  |  |  |  |  |  |  |  |
| car63 | .591 | .630 | .600 | .649 | .637 | 1.000 |  |  |  |  |  |  |  |  |
| car64 | .580 | .566 | .528 | .610 | .587 | .717 | 1.000 |  |  |  |  |  |  |  |
| enj67 | .337 | .369 | .273 | .373 | .346 | .495 | .553 | 1.000 |  |  |  |  |  |  |
| enj71 | .355 | .376 | .264 | .399 | .342 | .486 | .523 | .659 | 1.000 |  |  |  |  |  |
| enj74 | .332 | .392 | .349 | .429 | .364 | .491 | .532 | .641 | .758 | 1.000 |  |  |  |  |
| enj76 | .474 | .503 | .570 | .571 | .549 | .568 | .538 | .593 | .545 | .596 | 1.000 |  |  |  |
| enj77 | .351 | .356 | .302 | .382 | .357 | .469 | .456 | .585 | .594 | .605 | .591 | 1.000 |  |  |
| enj80 | .319 | .334 | .239 | .394 | .323 | .479 | .492 | .645 | .623 | .606 | .540 | .588 | 1.000 |  |
| enj81 | .342 | .388 | .277 | .384 | .345 | .534 | .537 | .660 | .701 | .668 | .563 | .625 | .737 | 1.000 |
| tra84 | .415 | .400 | .345 | .491 | .396 | .533 | .512 | .527 | .530 | .530 | .439 | .425 | .582 | .581 |
| tra86 | .483 | .525 | .471 | .563 | .505 | .620 | .588 | .453 | .466 | .461 | .507 | .380 | .488 | .463 |
| tra87 | .407 | .409 | .346 | .435 | .399 | .479 | .443 | .319 | .387 | .350 | .357 | .318 | .377 | .375 |
| tra89 | .465 | .452 | .450 | .499 | .487 | .526 | .513 | .489 | .474 | .493 | .558 | .464 | .493 | .528 |
| tra90 | .509 | .528 | .579 | .598 | .596 | .546 | .553 | .496 | .450 | .495 | .674 | .501 | .453 | .482 |
| tra96 | .368 | .361 | .318 | .401 | .394 | .446 | .407 | .403 | .410 | .374 | .371 | .316 | .402 | .389 |
| tra97 | .554 | .590 | .616 | .601 | .627 | .559 | .528 | .322 | .364 | .367 | .601 | .388 | .331 | .354 |

| **S8 Table. Polychoric correlations.** (Cont.) | | | | | | | |
| --- | --- | --- | --- | --- | --- | --- | --- |
|  | tra84 | tra86 | tra87 | tra89 | tra90 | tra96 | tra97 |
| tra84 | 1.000 |  |  |  |  |  |  |
| tra86 | .704 | 1.000 |  |  |  |  |  |
| tra87 | .608 | .719 | 1.000 |  |  |  |  |
| tra89 | .606 | .638 | .526 | 1.000 |  |  |  |
| tra90 | .490 | .595 | .523 | .681 | 1.000 |  |  |
| tra96 | .617 | .674 | .690 | .513 | .479 | 1.000 |  |
| tra97 | .435 | .577 | .529 | .556 | .690 | .519 | 1.000 |
