# Supplementary material for: Q-Herilearn: Assessing heritage learning in digital environments. A mixed approach with factor and IRT models
Source: PLoS One. 2024 Mar 29;19(3):e0299733. doi: 10.1371/journal.pone.0299733 (PMC10980239; doi:10.1371/journal.pone.0299733)
Supplement: S9 Table — (DOCX) [file pone.0299733.s009.docx]

| **S9 Table. Monte Carlo analysis results.** | | | | | | | |
| --- | --- | --- | --- | --- | --- | --- | --- |
| Parameter | Popul. | Aver. | Pop. SE | Aver. SE | MSE | 95%cover | 95%sig. |
| CARE ⇒ TRAN | .481 | .481 | .037 | .036 | .001 | .948 | 1.000 |
| ENJO ⇒ TRAN | .370 | .370 | .026 | .026 | .001 | .950 | 1.000 |
| KNOW ⇒ TRAN | .000 | .000 | .034 | .034 | .001 | .948 | .052 |
| RESP ⇒ TRAN | .000 | .000 | .036 | .037 | .001 | .952 | .048 |
| UNDE ⇒ TRAN | .000 | .000 | .038 | .038 | .001 | .947 | .053 |
| VALU ⇒ TRAN | .000 | .000 | .031 | .031 | .001 | .950 | .050 |
| KNOW ⇒ CARE | .327 | .327 | .023 | .023 | .001 | .949 | 1.000 |
| VALU ⇒ CARE | .321 | .321 | .021 | .021 | .000 | .952 | 1.000 |
| RESP ⇒ CARE | .000 | .000 | .028 | .028 | .001 | .947 | .053 |
| UNDE ⇒ CARE | .000 | .000 | .029 | .029 | .001 | .951 | .049 |
| CARE ⇒ ENJO | .210 | .210 | .038 | .038 | .001 | .951 | 1.000 |
| KNOW ⇒ ENJO | .368 | .371 | .033 | .034 | .001 | .952 | 1.000 |
| VALU ⇒ ENJO | .367 | .367 | .031 | .031 | .001 | .951 | 1.000 |
| RESP ⇒ ENJO | .000 | .000 | .038 | .038 | .002 | .950 | .050 |
| UNDE ⇒ ENJO | .000 | -.001 | .039 | .039 | .002 | .951 | .049 |
| UNDE ⇒ RESP | .591 | .591 | .023 | .023 | .001 | .949 | 1.000 |
| KNOW ⇒ RESP | .000 | .000 | .023 | .023 | .001 | .950 | .050 |
| RESP ⇒ VALU | .370 | .370 | .035 | .035 | .001 | .948 | 1.000 |
| KNOW ⇒ VALU | .187 | .187 | .030 | .029 | .001 | .948 | 1.000 |
| UNDE ⇒ VALU | .331 | .331 | .036 | .036 | .001 | .950 | 1.000 |
| KNOW ⇒ UNDE | .698 | .698 | .019 | .019 | .000 | .951 | 1.000 |
| *Note.* Popul. = Population; Aver. = Average of the estimated parameters in 10,000 replicates; SE | | | | | | | |
| = Standard Error of Estimation; MSE = Mean Squared Error; 95%cover = Proportion of replicates | | | | | | | |
| containing the population value according to a 95% confidence interval; 95%sig. = Proportion of | | | | | | | |
| replicates where the parameter was significant (p ≤ .05). | | | | |  |  |  |
