# Supplementary material for: Q-Herilearn: Assessing heritage learning in digital environments. A mixed approach with factor and IRT models
Source: PLoS One. 2024 Mar 29;19(3):e0299733. doi: 10.1371/journal.pone.0299733 (PMC10980239; doi:10.1371/journal.pone.0299733)
Supplement: S10 Table — (DOCX) [file pone.0299733.s010.docx]

| **S10 Table. Deleted participants and composition of the final sample.** | | | |
| --- | --- | --- | --- |
|  |  | **N** | **%** |
|  | Initial sample | 1454 | 100 |
| Deleted | Incomplete data | 65 | 4.47 |
|  | Straight lining | 9 | 0.62 |
|  | l^p^_z_ ( ≤ -3) | 26 | 1.79 |
|  | MV outliers | 26 | 1.79 |
|  | **Final sample** | **1328** | **91.33** |
