# Supplementary material for: Q-Herilearn: Assessing heritage learning in digital environments. A mixed approach with factor and IRT models
Source: PLoS One. 2024 Mar 29;19(3):e0299733. doi: 10.1371/journal.pone.0299733 (PMC10980239; doi:10.1371/journal.pone.0299733)
Supplement: S11 Table — (DOCX) [file pone.0299733.s011.docx]

| **S11 Table. Observed concordance matrix (Clarity).** | | | | |
| --- | --- | --- | --- | --- |
| Rating | 1 | 2 | 3 | 4 |
| 1 | 2.51 | 6.97 | 23.23 | 44.28 |
| 2 | 6.97 | 35.54 | 105.67 | 188.82 |
| 3 | 23.23 | 105.67 | 324.36 | 657.74 |
| 4 | 44.28 | 188.82 | 657.74 | 1464.15 |
