# Supplementary material for: Q-Herilearn: Assessing heritage learning in digital environments. A mixed approach with factor and IRT models
Source: PLoS One. 2024 Mar 29;19(3):e0299733. doi: 10.1371/journal.pone.0299733 (PMC10980239; doi:10.1371/journal.pone.0299733)
Supplement: S12 Table — (DOCX) [file pone.0299733.s012.docx]

| **S12 Table. Observed concordance matrix (Relevance).** | | | | |
| --- | --- | --- | --- | --- |
| Rating | 1 | 2 | 3 | 4 |
| 1 | 1.28 | 3.39 | 15.46 | 51.87 |
| 2 | 3.39 | 16.32 | 57.01 | 157.29 |
| 3 | 15.46 | 57.01 | 201.63 | 594.90 |
| 4 | 51.87 | 157.29 | 594.90 | 1898.94 |
