# Supplementary material for: Q-Herilearn: Assessing heritage learning in digital environments. A mixed approach with factor and IRT models
Source: PLoS One. 2024 Mar 29;19(3):e0299733. doi: 10.1371/journal.pone.0299733 (PMC10980239; doi:10.1371/journal.pone.0299733)
Supplement: S13 Table — (DOCX) [file pone.0299733.s013.docx]

| **S13 Table. Observed concordance matrix (Adequacy).** | | | | |
| --- | --- | --- | --- | --- |
| Rating | 1 | 2 | 3 | 4 |
| 1 | 0.67 | 4.21 | 15.82 | 45.30 |
| 2 | 4.21 | 17.74 | 62.98 | 160.07 |
| 3 | 15.82 | 62.98 | 241.49 | 622.71 |
| 4 | 45.30 | 160.07 | 622.71 | 1796.91 |
