# Supplementary material for: Q-Herilearn: Assessing heritage learning in digital environments. A mixed approach with factor and IRT models
Source: PLoS One. 2024 Mar 29;19(3):e0299733. doi: 10.1371/journal.pone.0299733 (PMC10980239; doi:10.1371/journal.pone.0299733)
Supplement: S14 Table — (DOCX) [file pone.0299733.s014.docx]

| **S14 Table. Observed concordance matrix (Dimension).** | | | | | | | |
| --- | --- | --- | --- | --- | --- | --- | --- |
| Dimension | Know | Unde | Resp | Valu | Care | Enjo | Tran |
| Know | 245.90 | 79.72 | 2.56 | 27.97 | 0.18 | 35.03 | 10.64 |
| Understand | 79.72 | 338.46 | 8.18 | 33.28 | 3.00 | 14.95 | 16.41 |
| Respect | 2.56 | 8.18 | 197.54 | 36.08 | 44.64 | 0.36 | 13.64 |
| Value | 27.97 | 33.28 | 36.08 | 439.64 | 33.10 | 16.46 | 47.46 |
| Care | 0.18 | 3.00 | 44.64 | 33.10 | 451.23 | 4.28 | 64.56 |
| Enjoy | 35.03 | 14.95 | 0.36 | 16.46 | 4.28 | 535.69 | 47.23 |
| Transmit | 10.64 | 16.41 | 13.64 | 47.46 | 64.56 | 47.23 | 592.05 |
