# Supplementary material for: Q-Herilearn: Assessing heritage learning in digital environments. A mixed approach with factor and IRT models
Source: PLoS One. 2024 Mar 29;19(3):e0299733. doi: 10.1371/journal.pone.0299733 (PMC10980239; doi:10.1371/journal.pone.0299733)
Supplement: S15 Table — (DOCX) [file pone.0299733.s015.docx]

| **S15 Table. Average Pairwise Proportional Agreement for Dimension.** | | | | | | | | | | | | | |
| --- | --- | --- | --- | --- | --- | --- | --- | --- | --- | --- | --- | --- | --- |
|  | n | 1 | 2 | 3 | 4 | 5 | 6 | 7 | 8 | 9 | 10 |  |  |
| n | rater | r02 | r04 | r06 | r16 | r22 | r23 | r26 | r27 | r28 | r29 |  |  |
| 1 | r02 | 1.000 |  |  |  |  |  |  |  |  |  |  |  |
| 2 | r04 | .753 | 1.000 |  |  |  |  |  |  |  |  |  |  |
| 3 | r06 | .825 | .773 | 1.000 |  |  |  |  |  |  |  |  |  |
| 4 | r16 | .825 | .732 | .907 | 1.000 |  |  |  |  |  |  |  |  |
| 5 | r22 | .691 | .608 | .691 | .691 | 1.000 |  |  |  |  |  |  |  |
| 6 | r23 | .680 | .619 | .691 | .722 | .773 | 1.000 |  |  |  |  |  |  |
| 7 | r26 | .794 | .742 | .918 | .887 | .680 | .660 | 1.000 |  |  |  |  |  |
| 8 | r27 | .835 | .763 | .907 | .928 | .680 | .711 | .887 | 1.000 |  |  |  |  |
| 9 | r28 | .753 | .608 | .711 | .701 | .670 | .598 | .742 | .670 | 1.000 |  |  |  |
| 10 | r29 | .773 | .742 | .897 | .876 | .670 | .701 | .907 | .876 | .722 | 1.000 |  |  |
| 11 | r30 | .742 | .639 | .722 | .753 | .670 | .670 | .742 | .722 | .619 | .711 |  |  |
| 12 | r31 | .773 | .794 | .856 | .835 | .680 | .680 | .835 | .835 | .680 | .887 |  |  |
| 13 | r32 | .608 | .619 | .629 | .608 | .495 | .443 | .619 | .619 | .588 | .608 |  |  |
| 14 | r39 | .340 | .371 | .392 | .412 | .392 | .361 | .381 | .371 | .340 | .433 |  |  |
| 15 | r40 | .804 | .722 | .918 | .918 | .680 | .691 | .876 | .907 | .722 | .876 |  |  |
| 16 | r42 | .773 | .680 | .814 | .804 | .639 | .598 | .825 | .794 | .701 | .763 |  |  |
| 17 | r43 | .649 | .639 | .660 | .670 | .639 | .608 | .691 | .649 | .649 | .701 |  |  |
| 18 | r45 | .784 | .753 | .876 | .866 | .691 | .722 | .887 | .897 | .680 | .897 |  |  |
| 19 | r46 | .825 | .732 | .907 | .938 | .701 | .670 | .887 | .918 | .742 | .876 |  |  |
| 20 | r47 | .763 | .732 | .794 | .814 | .691 | .649 | .753 | .773 | .701 | .763 |  |  |
| 21 | r50 | .732 | .742 | .773 | .753 | .577 | .577 | .784 | .753 | .639 | .732 |  |  |
| 22 | r53 | .794 | .680 | .794 | .856 | .680 | .670 | .804 | .825 | .711 | .794 |  |  |
| 23 | r54 | .639 | .629 | .670 | .701 | .598 | .598 | .670 | .691 | .546 | .680 |  |  |
| 24 | r55 | .753 | .701 | .814 | .814 | .660 | .680 | .825 | .835 | .680 | .794 |  |  |
| 25 | r56 | .814 | .794 | .814 | .804 | .701 | .701 | .825 | .814 | .753 | .825 |  |  |
| 26 | r57 | .794 | .794 | .814 | .804 | .701 | .701 | .825 | .814 | .742 | .825 |  |  |
| 27 | r60 | .804 | .794 | .845 | .825 | .691 | .691 | .856 | .845 | .742 | .856 |  |  |
| 28 | r61 | .794 | .732 | .866 | .845 | .691 | .701 | .814 | .876 | .629 | .825 |  |  |
| 29 | r62 | .412 | .443 | .423 | .433 | .381 | .351 | .423 | .412 | .443 | .423 |  |  |
| 30 | r63 | .742 | .753 | .835 | .835 | .691 | .660 | .835 | .804 | .660 | .814 |  | .00 |
| 31 | r65 | .814 | .742 | .928 | .866 | .670 | .670 | .866 | .876 | .711 | .856 |  | .10 |
| 32 | r67 | .773 | .722 | .876 | .866 | .660 | .660 | .856 | .866 | .711 | .876 |  | .20 |
| 33 | r70 | .804 | .732 | .866 | .876 | .691 | .660 | .866 | .897 | .701 | .814 |  | .30 |
| 34 | r72 | .825 | .691 | .814 | .814 | .619 | .588 | .773 | .804 | .660 | .763 |  | .40 |
| 35 | r76 | .742 | .753 | .835 | .845 | .691 | .680 | .825 | .814 | .649 | .856 |  | .50 |
| 36 | r78 | .845 | .753 | .918 | .918 | .701 | .691 | .907 | .907 | .742 | .856 |  | .60 |
| 37 | r80 | .732 | .680 | .794 | .773 | .588 | .639 | .794 | .825 | .670 | .804 |  | .70 |
| 38 | r82 | .763 | .680 | .835 | .835 | .711 | .701 | .814 | .845 | .608 | .845 |  | .80 |
| 39 | r84 | .825 | .784 | .938 | .897 | .691 | .711 | .876 | .928 | .691 | .866 |  | .90 |
| 40 | r86 | .814 | .732 | .938 | .918 | .691 | .691 | .897 | .928 | .732 | .897 |  | 1.00 |

| **S15 Table. Average Pairwise Proportional Agreement for Dimension.** (Cont.) | | | | | | | | | | | | | |
| --- | --- | --- | --- | --- | --- | --- | --- | --- | --- | --- | --- | --- | --- |
|  | n | 11 | 12 | 13 | 14 | 15 | 16 | 17 | 18 | 19 | 20 |  |  |
| n | rater | r30 | r31 | r32 | r39 | r40 | r42 | r43 | r45 | r46 | r47 |  |  |
| 11 | r30 | 1.000 |  |  |  |  |  |  |  |  |  |  |  |
| 12 | r31 | .711 | 1.000 |  |  |  |  |  |  |  |  |  |  |
| 13 | r32 | .546 | .649 | 1.000 |  |  |  |  |  |  |  |  |  |
| 14 | r39 | .402 | .412 | .351 | 1.000 |  |  |  |  |  |  |  |  |
| 15 | r40 | .691 | .825 | .608 | .402 | 1.000 |  |  |  |  |  |  |  |
| 16 | r42 | .660 | .742 | .598 | .330 | .784 | 1.000 |  |  |  |  |  |  |
| 17 | r43 | .639 | .660 | .526 | .381 | .629 | .577 | 1.000 |  |  |  |  |  |
| 18 | r45 | .742 | .845 | .608 | .412 | .876 | .753 | .660 | 1.000 |  |  |  |  |
| 19 | r46 | .722 | .845 | .619 | .402 | .948 | .814 | .639 | .876 | 1.000 |  |  |  |
| 20 | r47 | .639 | .742 | .567 | .392 | .814 | .732 | .670 | .742 | .814 | 1.000 |  |  |
| 21 | r50 | .608 | .701 | .567 | .330 | .763 | .691 | .629 | .732 | .722 | .753 |  |  |
| 22 | r53 | .784 | .763 | .588 | .433 | .784 | .742 | .629 | .856 | .814 | .732 |  |  |
| 23 | r54 | .567 | .629 | .464 | .340 | .691 | .629 | .660 | .639 | .670 | .629 |  |  |
| 24 | r55 | .680 | .742 | .526 | .340 | .804 | .691 | .660 | .814 | .773 | .680 |  |  |
| 25 | r56 | .732 | .835 | .660 | .361 | .773 | .753 | .711 | .825 | .784 | .742 |  |  |
| 26 | r57 | .711 | .835 | .649 | .371 | .784 | .732 | .701 | .825 | .794 | .732 |  |  |
| 27 | r60 | .722 | .835 | .660 | .392 | .814 | .753 | .732 | .856 | .814 | .742 |  |  |
| 28 | r61 | .701 | .784 | .598 | .361 | .835 | .732 | .619 | .856 | .835 | .753 |  |  |
| 29 | r62 | .361 | .402 | .423 | .330 | .433 | .392 | .371 | .443 | .433 | .474 |  |  |
| 30 | r63 | .711 | .845 | .608 | .392 | .794 | .722 | .629 | .814 | .794 | .722 |  | .00 |
| 31 | r65 | .660 | .825 | .629 | .371 | .897 | .804 | .639 | .845 | .876 | .773 |  | .10 |
| 32 | r67 | .691 | .856 | .619 | .412 | .907 | .742 | .608 | .866 | .907 | .763 |  | .20 |
| 33 | r70 | .680 | .814 | .629 | .381 | .876 | .804 | .629 | .814 | .897 | .784 |  | .30 |
| 34 | r72 | .691 | .763 | .619 | .381 | .804 | .784 | .608 | .794 | .814 | .732 |  | .40 |
| 35 | r76 | .722 | .835 | .577 | .423 | .794 | .722 | .639 | .814 | .825 | .711 |  | .50 |
| 36 | r78 | .722 | .835 | .639 | .371 | .907 | .835 | .680 | .856 | .907 | .794 |  | .60 |
| 37 | r80 | .639 | .753 | .567 | .392 | .784 | .639 | .619 | .835 | .773 | .660 |  | .70 |
| 38 | r82 | .701 | .784 | .515 | .392 | .835 | .701 | .722 | .804 | .845 | .722 |  | .80 |
| 39 | r84 | .722 | .856 | .649 | .381 | .897 | .784 | .670 | .918 | .887 | .763 |  | .90 |
| 40 | r86 | .711 | .845 | .608 | .392 | .979 | .794 | .629 | .897 | .969 | .804 |  | 1.00 |

| **S15 Table. Average Pairwise Proportional Agreement for Dimension.** (Cont.) | | | | | | | | | | | | | |
| --- | --- | --- | --- | --- | --- | --- | --- | --- | --- | --- | --- | --- | --- |
|  | n | 21 | 22 | 23 | 24 | 25 | 26 | 27 | 28 | 29 | 30 |  |  |
| n | rater | r50 | r53 | r54 | r55 | r56 | r57 | r60 | r61 | r62 | r63 |  |  |
| 21 | r50 | 1.000 |  |  |  |  |  |  |  |  |  |  |  |
| 22 | r53 | .701 | 1.000 |  |  |  |  |  |  |  |  |  |  |
| 23 | r54 | .608 | .619 | 1.000 |  |  |  |  |  |  |  |  |  |
| 24 | r55 | .742 | .732 | .680 | 1.000 |  |  |  |  |  |  |  |  |
| 25 | r56 | .742 | .804 | .649 | .773 | 1.000 |  |  |  |  |  |  |  |
| 26 | r57 | .722 | .784 | .660 | .773 | .979 | 1.000 |  |  |  |  |  |  |
| 27 | r60 | .753 | .804 | .680 | .804 | .948 | .969 | 1.000 |  |  |  |  |  |
| 28 | r61 | .711 | .784 | .660 | .773 | .794 | .794 | .825 | 1.000 |  |  |  |  |
| 29 | r62 | .412 | .464 | .381 | .402 | .402 | .412 | .433 | .443 | 1.000 |  |  |  |
| 30 | r63 | .691 | .763 | .619 | .763 | .825 | .825 | .814 | .814 | .433 | 1.000 |  | .00 |
| 31 | r65 | .753 | .763 | .660 | .773 | .794 | .794 | .825 | .835 | .454 | .784 |  | .10 |
| 32 | r67 | .701 | .773 | .649 | .773 | .804 | .814 | .804 | .794 | .433 | .794 |  | .20 |
| 33 | r70 | .753 | .784 | .639 | .753 | .784 | .794 | .814 | .784 | .433 | .773 |  | .30 |
| 34 | r72 | .701 | .773 | .629 | .680 | .753 | .732 | .753 | .784 | .412 | .711 |  | .40 |
| 35 | r76 | .660 | .763 | .670 | .763 | .804 | .804 | .804 | .794 | .402 | .845 |  | .50 |
| 36 | r78 | .773 | .825 | .670 | .804 | .825 | .825 | .856 | .825 | .412 | .804 |  | .60 |
| 37 | r80 | .670 | .753 | .577 | .753 | .773 | .773 | .804 | .804 | .412 | .742 |  | .70 |
| 38 | r82 | .670 | .732 | .722 | .732 | .691 | .691 | .722 | .763 | .361 | .701 |  | .80 |
| 39 | r84 | .773 | .845 | .649 | .814 | .835 | .835 | .866 | .897 | .433 | .835 |  | .90 |
| 40 | r86 | .742 | .804 | .670 | .804 | .784 | .794 | .825 | .856 | .433 | .794 |  | 1.00 |

| **S15 Table. Average Pairwise Proportional Agreement for Dimension.** (Cont.) | | | | | | | | | | | | | |
| --- | --- | --- | --- | --- | --- | --- | --- | --- | --- | --- | --- | --- | --- |
|  | n | 31 | 32 | 33 | 34 | 35 | 36 | 37 | 38 | 39 | 40 |  |  |
| n | rater | r65 | r67 | r70 | r72 | r76 | r78 | r80 | r82 | r84 | r86 |  | .00 |
| 31 | r65 | 1.000 |  |  |  |  |  |  |  |  |  |  | .10 |
| 32 | r67 | .856 | 1.000 |  |  |  |  |  |  |  |  |  | .20 |
| 33 | r70 | .856 | .845 | 1.000 |  |  |  |  |  |  |  |  | .30 |
| 34 | r72 | .814 | .753 | .763 | 1.000 |  |  |  |  |  |  |  | .40 |
| 35 | r76 | .784 | .804 | .763 | .722 | 1.000 |  |  |  |  |  |  | .50 |
| 36 | r78 | .907 | .856 | .907 | .814 | .804 | 1.000 |  |  |  |  |  | .60 |
| 37 | r80 | .784 | .794 | .742 | .701 | .722 | .784 | 1.000 |  |  |  |  | .70 |
| 38 | r82 | .804 | .794 | .804 | .711 | .753 | .814 | .691 | 1.000 |  |  |  | .80 |
| 39 | r84 | .907 | .856 | .866 | .835 | .804 | .918 | .856 | .814 | 1.000 |  |  | .90 |
| 40 | r86 | .907 | .928 | .887 | .804 | .814 | .918 | .804 | .856 | .918 | 1.000 |  | 1.00 |
