# Supplementary material for: Q-Herilearn: Assessing heritage learning in digital environments. A mixed approach with factor and IRT models
Source: PLoS One. 2024 Mar 29;19(3):e0299733. doi: 10.1371/journal.pone.0299733 (PMC10980239; doi:10.1371/journal.pone.0299733)
Supplement: S16 Table — (DOCX) [file pone.0299733.s016.docx]

| **S16 Table. Average Pairwise Cohen's Kappa for Dimension.** | | | | | | | | | | | | | |
| --- | --- | --- | --- | --- | --- | --- | --- | --- | --- | --- | --- | --- | --- |
|  | n | 1 | 2 | 3 | 4 | 5 | 6 | 7 | 8 | 9 | 10 |  |  |
| n | rater | r02 | r04 | r06 | r16 | r22 | r23 | r26 | r27 | r28 | r29 |  |  |
| 1 | r02 | 1.000 |  |  |  |  |  |  |  |  |  |  |  |
| 2 | r04 | .703 | 1.000 |  |  |  |  |  |  |  |  |  |  |
| 3 | r06 | .794 | .731 | 1.000 |  |  |  |  |  |  |  |  |  |
| 4 | r16 | .793 | .681 | .891 | 1.000 |  |  |  |  |  |  |  |  |
| 5 | r22 | .633 | .531 | .636 | .635 | 1.000 |  |  |  |  |  |  |  |
| 6 | r23 | .622 | .541 | .636 | .672 | .730 | 1.000 |  |  |  |  |  |  |
| 7 | r26 | .757 | .694 | .903 | .867 | .622 | .599 | 1.000 |  |  |  |  |  |
| 8 | r27 | .806 | .718 | .891 | .915 | .624 | .658 | .867 | 1.000 |  |  |  |  |
| 9 | r28 | .706 | .533 | .662 | .648 | .607 | .528 | .696 | .614 | 1.000 |  |  |  |
| 10 | r29 | .734 | .694 | .879 | .855 | .610 | .645 | .891 | .855 | .673 | 1.000 |  |  |
| 11 | r30 | .692 | .563 | .672 | .707 | .604 | .605 | .695 | .671 | .546 | .658 |  |  |
| 12 | r31 | .732 | .754 | .830 | .806 | .622 | .621 | .806 | .806 | .624 | .867 |  |  |
| 13 | r32 | .531 | .543 | .565 | .538 | .408 | .350 | .552 | .552 | .509 | .543 |  |  |
| 14 | r39 | .227 | .255 | .287 | .311 | .278 | .242 | .273 | .263 | .226 | .331 |  |  |
| 15 | r40 | .770 | .672 | .903 | .903 | .624 | .636 | .855 | .891 | .674 | .855 |  |  |
| 16 | r42 | .733 | .623 | .783 | .770 | .574 | .531 | .794 | .759 | .648 | .723 |  |  |
| 17 | r43 | .580 | .560 | .599 | .608 | .562 | .529 | .632 | .587 | .575 | .643 |  |  |
| 18 | r45 | .746 | .705 | .855 | .843 | .637 | .670 | .867 | .879 | .627 | .879 |  |  |
| 19 | r46 | .794 | .684 | .891 | .927 | .648 | .612 | .867 | .903 | .698 | .855 |  |  |
| 20 | r47 | .716 | .676 | .757 | .780 | .633 | .583 | .708 | .732 | .644 | .721 |  |  |
| 21 | r50 | .682 | .688 | .733 | .708 | .501 | .501 | .744 | .708 | .572 | .684 |  |  |
| 22 | r53 | .756 | .616 | .758 | .830 | .621 | .610 | .769 | .794 | .659 | .757 |  |  |
| 23 | r54 | .570 | .550 | .611 | .645 | .507 | .511 | .607 | .635 | .452 | .616 |  |  |
| 24 | r55 | .710 | .644 | .782 | .782 | .597 | .619 | .794 | .806 | .624 | .756 |  |  |
| 25 | r56 | .780 | .751 | .781 | .769 | .644 | .646 | .793 | .781 | .707 | .793 |  |  |
| 26 | r57 | .755 | .751 | .781 | .769 | .644 | .645 | .793 | .781 | .695 | .793 |  |  |
| 27 | r60 | .768 | .752 | .818 | .793 | .633 | .632 | .830 | .818 | .696 | .830 |  |  |
| 28 | r61 | .756 | .680 | .842 | .818 | .636 | .645 | .782 | .854 | .566 | .794 |  |  |
| 29 | r62 | .292 | .317 | .320 | .326 | .252 | .219 | .314 | .307 | .322 | .312 |  |  |
| 30 | r63 | .693 | .701 | .805 | .805 | .630 | .596 | .805 | .769 | .597 | .781 |  | .00 |
| 31 | r65 | .781 | .693 | .915 | .843 | .611 | .610 | .843 | .855 | .660 | .830 |  | .10 |
| 32 | r67 | .734 | .672 | .855 | .843 | .599 | .598 | .831 | .843 | .663 | .855 |  | .20 |
| 33 | r70 | .768 | .680 | .843 | .854 | .635 | .600 | .842 | .879 | .647 | .782 |  | .30 |
| 34 | r72 | .791 | .629 | .782 | .781 | .550 | .513 | .733 | .769 | .599 | .722 |  | .40 |
| 35 | r76 | .695 | .702 | .806 | .817 | .629 | .618 | .793 | .781 | .584 | .829 |  | .50 |
| 36 | r78 | .817 | .706 | .903 | .903 | .648 | .636 | .891 | .891 | .696 | .831 |  | .60 |
| 37 | r80 | .686 | .620 | .759 | .735 | .520 | .573 | .759 | .794 | .615 | .770 |  | .70 |
| 38 | r82 | .721 | .620 | .806 | .806 | .654 | .642 | .781 | .818 | .536 | .817 |  | .80 |
| 39 | r84 | .793 | .742 | .927 | .879 | .637 | .659 | .855 | .915 | .638 | .843 |  | .90 |
| 40 | r86 | .783 | .685 | .928 | .903 | .637 | .635 | .879 | .915 | .687 | .879 |  | 1.00 |

| **S16 Table. Average Pairwise Cohen's Kappa for Dimension.** (Cont.) | | | | | | | | | | | | | |
| --- | --- | --- | --- | --- | --- | --- | --- | --- | --- | --- | --- | --- | --- |
|  | n | 11 | 12 | 13 | 14 | 15 | 16 | 17 | 18 | 19 | 20 |  |  |
| n | rater | r30 | r31 | r32 | r39 | r40 | r42 | r43 | r45 | r46 | r47 |  |  |
| 11 | r30 | 1.000 |  |  |  |  |  |  |  |  |  |  |  |
| 12 | r31 | .657 | 1.000 |  |  |  |  |  |  |  |  |  |  |
| 13 | r32 | .463 | .587 | 1.000 |  |  |  |  |  |  |  |  |  |
| 14 | r39 | .288 | .309 | .245 | 1.000 |  |  |  |  |  |  |  |  |
| 15 | r40 | .635 | .794 | .541 | .298 | 1.000 |  |  |  |  |  |  |  |
| 16 | r42 | .599 | .698 | .527 | .218 | .747 | 1.000 |  |  |  |  |  |  |
| 17 | r43 | .561 | .595 | .437 | .261 | .562 | .503 | 1.000 |  |  |  |  |  |
| 18 | r45 | .696 | .818 | .542 | .310 | .855 | .711 | .600 | 1.000 |  |  |  |  |
| 19 | r46 | .672 | .819 | .553 | .299 | .940 | .783 | .574 | .855 | 1.000 |  |  |  |
| 20 | r47 | .568 | .695 | .481 | .285 | .782 | .685 | .603 | .697 | .782 | 1.000 |  |  |
| 21 | r50 | .536 | .647 | .484 | .218 | .723 | .636 | .557 | .683 | .674 | .705 |  |  |
| 22 | r53 | .742 | .720 | .513 | .332 | .746 | .697 | .556 | .830 | .782 | .682 |  |  |
| 23 | r54 | .470 | .558 | .375 | .201 | .634 | .564 | .571 | .575 | .610 | .556 |  |  |
| 24 | r55 | .621 | .697 | .447 | .221 | .770 | .639 | .592 | .782 | .735 | .623 |  |  |
| 25 | r56 | .680 | .805 | .597 | .248 | .734 | .708 | .654 | .793 | .746 | .693 |  |  |
| 26 | r57 | .655 | .805 | .586 | .259 | .746 | .684 | .641 | .793 | .758 | .681 |  |  |
| 27 | r60 | .669 | .805 | .600 | .283 | .782 | .709 | .679 | .830 | .782 | .694 |  |  |
| 28 | r61 | .646 | .745 | .526 | .251 | .806 | .687 | .549 | .830 | .807 | .706 |  |  |
| 29 | r62 | .223 | .288 | .307 | .200 | .332 | .286 | .212 | .345 | .332 | .363 |  |  |
| 30 | r63 | .653 | .816 | .536 | .283 | .757 | .670 | .555 | .781 | .757 | .667 |  | .00 |
| 31 | r65 | .597 | .794 | .564 | .261 | .879 | .771 | .570 | .818 | .855 | .732 |  | .10 |
| 32 | r67 | .635 | .831 | .555 | .310 | .891 | .699 | .538 | .843 | .891 | .721 |  | .20 |
| 33 | r70 | .622 | .782 | .560 | .276 | .855 | .770 | .559 | .782 | .879 | .743 |  | .30 |
| 34 | r72 | .632 | .720 | .545 | .275 | .770 | .746 | .536 | .757 | .782 | .680 |  | .40 |
| 35 | r76 | .666 | .805 | .505 | .314 | .758 | .673 | .562 | .781 | .794 | .657 |  | .50 |
| 36 | r78 | .671 | .806 | .574 | .264 | .891 | .807 | .621 | .831 | .891 | .756 |  | .60 |
| 37 | r80 | .577 | .710 | .492 | .288 | .747 | .582 | .550 | .806 | .736 | .600 |  | .70 |
| 38 | r82 | .643 | .745 | .437 | .277 | .806 | .650 | .663 | .770 | .818 | .671 |  | .80 |
| 39 | r84 | .672 | .830 | .586 | .277 | .879 | .747 | .612 | .903 | .867 | .719 |  | .90 |
| 40 | r86 | .660 | .819 | .543 | .286 | .976 | .759 | .564 | .879 | .964 | .770 |  | 1.00 |

| **S16 Table. Average Pairwise Cohen's Kappa for Dimension.** (Cont.) | | | | | | | | | | | | | |
| --- | --- | --- | --- | --- | --- | --- | --- | --- | --- | --- | --- | --- | --- |
|  | n | 21 | 22 | 23 | 24 | 25 | 26 | 27 | 28 | 29 | 30 |  |  |
| n | rater | r50 | r53 | r54 | r55 | r56 | r57 | r60 | r61 | r62 | r63 |  |  |
| 21 | r50 | 1.000 |  |  |  |  |  |  |  |  |  |  |  |
| 22 | r53 | .645 | 1.000 |  |  |  |  |  |  |  |  |  |  |
| 23 | r54 | .537 | .544 | 1.000 |  |  |  |  |  |  |  |  |  |
| 24 | r55 | .696 | .684 | .614 | 1.000 |  |  |  |  |  |  |  |  |
| 25 | r56 | .692 | .767 | .581 | .733 | 1.000 |  |  |  |  |  |  |  |
| 26 | r57 | .668 | .743 | .591 | .732 | .975 | 1.000 |  |  |  |  |  |  |
| 27 | r60 | .705 | .768 | .617 | .768 | .939 | .963 | 1.000 |  |  |  |  |  |
| 28 | r61 | .659 | .745 | .599 | .733 | .757 | .756 | .793 | 1.000 |  |  |  |  |
| 29 | r62 | .295 | .358 | .222 | .283 | .283 | .294 | .321 | .340 | 1.000 |  |  |  |
| 30 | r63 | .632 | .718 | .542 | .721 | .790 | .790 | .779 | .780 | .322 | 1.000 |  | .00 |
| 31 | r65 | .707 | .720 | .595 | .733 | .756 | .756 | .793 | .806 | .348 | .745 |  | .10 |
| 32 | r67 | .650 | .734 | .585 | .733 | .770 | .782 | .770 | .758 | .332 | .757 |  | .20 |
| 33 | r70 | .707 | .745 | .573 | .710 | .744 | .756 | .781 | .745 | .325 | .731 |  | .30 |
| 34 | r72 | .646 | .732 | .563 | .626 | .707 | .682 | .708 | .744 | .300 | .656 |  | .40 |
| 35 | r76 | .597 | .718 | .596 | .718 | .767 | .766 | .767 | .757 | .276 | .815 |  | .50 |
| 36 | r78 | .732 | .794 | .610 | .770 | .793 | .793 | .830 | .794 | .302 | .768 |  | .60 |
| 37 | r80 | .609 | .710 | .503 | .708 | .734 | .734 | .769 | .769 | .302 | .700 |  | .70 |
| 38 | r82 | .613 | .684 | .657 | .681 | .635 | .634 | .671 | .721 | .229 | .646 |  | .80 |
| 39 | r84 | .731 | .818 | .589 | .782 | .805 | .805 | .842 | .878 | .331 | .805 |  | .90 |
| 40 | r86 | .699 | .771 | .611 | .770 | .746 | .758 | .795 | .831 | .334 | .758 |  | 1.00 |

| **S16 Table. Average Pairwise Cohen's Kappa for Dimension.** (Cont.) | | | | | | | | | | | | | |
| --- | --- | --- | --- | --- | --- | --- | --- | --- | --- | --- | --- | --- | --- |
|  | n | 31 | 32 | 33 | 34 | 35 | 36 | 37 | 38 | 39 | 40 |  |  |
| n | rater | r65 | r67 | r70 | r72 | r76 | r78 | r80 | r82 | r84 | r86 |  | .00 |
| 31 | r65 | 1.000 |  |  |  |  |  |  |  |  |  |  | .10 |
| 32 | r67 | .831 | 1.000 |  |  |  |  |  |  |  |  |  | .20 |
| 33 | r70 | .830 | .819 | 1.000 |  |  |  |  |  |  |  |  | .30 |
| 34 | r72 | .782 | .710 | .720 | 1.000 |  |  |  |  |  |  |  | .40 |
| 35 | r76 | .744 | .769 | .720 | .672 | 1.000 |  |  |  |  |  |  | .50 |
| 36 | r78 | .891 | .831 | .891 | .781 | .769 | 1.000 |  |  |  |  |  | .60 |
| 37 | r80 | .745 | .759 | .698 | .650 | .674 | .746 | 1.000 |  |  |  |  | .70 |
| 38 | r82 | .769 | .757 | .770 | .662 | .704 | .782 | .638 | 1.000 |  |  |  | .80 |
| 39 | r84 | .891 | .831 | .842 | .805 | .770 | .903 | .830 | .783 | 1.000 |  |  | .90 |
| 40 | r86 | .891 | .915 | .867 | .770 | .782 | .904 | .771 | .830 | .903 | 1.000 |  | 1.00 |
