# Supplementary material for: Q-Herilearn: Assessing heritage learning in digital environments. A mixed approach with factor and IRT models
Source: PLoS One. 2024 Mar 29;19(3):e0299733. doi: 10.1371/journal.pone.0299733 (PMC10980239; doi:10.1371/journal.pone.0299733)
Supplement: S17 Table — (DOCX) [file pone.0299733.s017.docx]

| **S17 Table. Summary of Inter-rater Agreement analysis for Dimension.** | | | | | | |
| --- | --- | --- | --- | --- | --- | --- |
| κ | OA | EA | α | ND | Σ_c_O_cc_ | Σ_c_n_c_(n_c_-1) |
| .671 | .722 | .154 | .671 | 3880 | 2800.51 | 2311706.00 |
| *Note.* κ = Fleiss' kappa; OA = Observed Agreement; EA = Expected | | | | | | |
| Agreement; α = Krippendorff's alpha. | | | | | | |
