# Supplementary material for: Q-Herilearn: Assessing heritage learning in digital environments. A mixed approach with factor and IRT models
Source: PLoS One. 2024 Mar 29;19(3):e0299733. doi: 10.1371/journal.pone.0299733 (PMC10980239; doi:10.1371/journal.pone.0299733)
Supplement: S18 Table — (DOCX) [file pone.0299733.s018.docx]

| **S18 Table. Inter-factor correlations.** | | | | | | | |
| --- | --- | --- | --- | --- | --- | --- | --- |
|  | KNO | UND | RES | VAL | CAR | ENJ | TRA |
| KNO | 1.000 |  |  |  |  |  |  |
| UND | .503 | 1.000 |  |  |  |  |  |
| RES | .225 | .482 | 1.000 |  |  |  |  |
| VAL | .435 | .602 | .491 | 1.000 |  |  |  |
| CAR | .364 | .197 | -.075 | .352 | 1.000 |  |  |
| ENJ | .570 | .494 | .428 | .586 | .461 | 1.000 |  |
| TRA | .382 | .339 | .188 | .481 | .536 | .568 | 1.000 |
